# How far is the journey before malaria is knocked out malaria in Zimbabwe: results of the malaria indicator survey 2016

**DOI:** 10.1186/s12936-019-2801-3

**Published:** 2019-05-14

**Authors:** Busisani Dube, Joseph Mberikunashe, Patience Dhliwayo, Andrew Tangwena, Gerald Shambira, Anderson Chimusoro, Munashe Madinga, Brighton Gambinga

**Affiliations:** 1 National Malaria Control Programme, Harare, Zimbabwe; 2 University of Zimbabwe, College of Health Sciences, Harare, Zimbabwe; 3 World Health Organization, Country Office, Harare, Zimbabwe; 4 Clinton Health Access Initiative, Country Office, Harare, Zimbabwe

**Keywords:** Malaria, Zimbabwe, Elimination, Vector control, Indicator, Survey, Enumeration area

## Abstract

**Background:**

Zimbabwe conducts Malaria Indicator Surveys after 3 years and Demographic and Health Surveys to track the impact of malaria interventions. The last one to be conducted was in 2016 and had set an aim aimed to collect data to track malaria indicators as well as to save as the baseline source for the Malaria Strategic Plan (2016–2020).

**Methods:**

Malaria Indicator Survey-2016 utilized the frame of enumeration areas (EAs) from the Zimbabwe Master Sample (ZMS12) created after the 2012 population census for each of the survey districts. The design for the survey was a representative probability sample to produce estimates at national level for the respective domains, which are the forty-four malaria-endemic districts. Survey teams comprised of Ministry of Health personnel who administered the standard questionnaire (adapted to country setting) to respondents within sampled EAs, performed RDT, anaemia test, prepared microscopic slide and collected DBS and data analysis of collected information was analysed. Microscopic slides examined centrally at the National Institute of Health Research.

**Results:**

The overall protection coverage by at least one major vector control measure, IRS and/or Nets, was 82.5%. Use of nets among high-risk groups 32.5% For children under five and 24.5% for pregnant women. LLIN utilization quite low taking into consideration the net ownership per household, which was 58% for the general population. Moreover, IPTp coverage has remained almost unchanged since the 2012 MIS, with only a third of pregnant women receiving at least two doses of IPTp. Malaria prevalence appears to be on the decline with 2016 MIS recording 0.2% compared to 0.4% as of 2012 MIS. *Plasmodium falciparum* remains the predominant parasite species in the country at 98%.

**Conclusion:**

The results indicated that some progress has been made in malaria control although there is still subsequent low malaria risk perception that comes with the reduced prevalence. It has been shown that there is low use of interventions shown by the low use of LLINs by vulnerable groups like pregnant women and children under five.

## Background

There are 60 rural districts in the country of which 51 are malarious with varying transmission intensity. Of the malarious districts, 45 are in the low-lying areas of the country characterized by high temperatures (up to 39 C). During the peak malaria transmission season, sporadic epidemics are reported in the high burdened districts. The population affected varies from year to year depending on the performance and coverage of the various malaria prevention and control interventions carried out by the National Malaria Control Programme. Apart from the routine health information system, Zimbabwe conducts regular surveys to measure some indicators whose data may not be sufficiently collected in routine health information. Zimbabwe Demographic and Health Surveys (ZDHS) and Multiple Indicator Cluster Surveys (MICS) collect information on some malaria indicators. The first malaria indicator survey was carried out in 2008 and the second was carried out in 2012 on a national scale following the approach of the MICS and the ZDHS. The ZDHS 2011/2012 indicated significant progress in the coverage of LLINs and high IRS coverage.

The NMCP in collaboration with multiple partners sets high targets for coverage of interventions and reduction in malaria burden as outlined in the National Malaria Strategy 2008–2015. The major anti-malaria strategies focus on malaria prevention, case management, detection and control of epidemics, social and behavior change communication (SBCC) and surveillance, monitoring and evaluation, and operational research [1]. IRS with insecticide and the distribution of LLINs are the major malaria prevention measures targeted at areas with ongoing malaria transmission. SP/Fansidar is given to all eligible pregnant women residing in high-burden areas, starting as soon as possible after the first trimester and continuing every four weeks thereafter until delivery [2, 3]. Case management is offered at all health facilities in the country. Treatment of uncomplicated malaria is based on artemisinin-based combination therapy (ACT); the first-line treatment is artemether-lumefantrine (Coartem®), while the second line is artesunate–amodiaquine. An alternative second line treatment is oral quinine in combination with either doxycycline or clindamycin, or oral quinine alone in children under 8 years of age.

The data collection for these surveys does not necessarily consider the peak transmission season, hence the need for the country to carry out the malaria indicator survey to focus on the malaria transmission areas and collect relevant data on coverage of key malaria interventions and related results during the peak transmission season. The nationwide malaria indicator survey set to have malaria baseline data for the next 5-year Malaria Strategic Plan after the expiration of the current one. It also had an objective to collect data that will be used for the re-stratification of the country’s malaria transmission zones.

## Methods

### Survey organization

The 2016 Zimbabwe Malaria Indicator Survey was implemented NMCP in cooperation with the Zimbabwe National Statistics Agency (ZIMSTAT) and other malaria partners in the country. ZIMSTAT was responsible for general administrative management of the survey, including overseeing the day-to-day operations, designing the survey, and processing the data. ZIMSTAT also assisted NMCP in the design of the survey, especially in the area of sample design and selection, where they provided the necessary maps and lists of households in the selected sample points. The NMCP took primary responsibility for organizing the Technical Working Group, developed the survey protocol in preparation of ethical review in preparation of ethical review, by the Medical Research Council of Zimbabwe. Training was conducted by ZIMSTAT and NMCP while Provincial Medical Directors (PMD) oversaw the recruitment of field staff with support from key malaria partners. Medicines to treat under-fives who tested positive for malaria during the survey were provided by the MOHCC. Technical assistance was provided by various partners including NIHR, WHO, CHAI, PSI and ZAPIM. Financial support for the survey was provided by the Government of Zimbabwe Global Fund and U.S. President’s Malaria Initiative (PMI) through the Zimbabwe Assistance Program in Malaria (ZAPIM).

### Sample design

Zimbabwe is administratively divided into eight rural provinces (Manicaland, Mashonaland Central, Mashonaland East, Mashonaland West, Matabeleland North, Matabeleland South, Midlands and Masvingo) and two urban provinces, which are all in turn subdivided into districts. Each district is further subdivided into wards. For the MIS 2016, wards were subdivided into enumeration areas (EA), which is the smallest working unit in any census or survey operation that can be easily covered by an interviewer or team in 1 day. The 2016 MIS utilized the EA frame from the Zimbabwe Master Sample (ZMS12), created after the 2012 Population Census for each of the survey districts. The MIS only covered households in the 45 designated malaria endemic districts and these districts were further divided into moderate and high malaria transmission zones based on routine data from the NMCP.

The overall design of the survey was based on a representative probability sample to produce estimates at transmission level for the respective domains, which are the 45 malaria endemic districts in the survey [4]. All women aged 15–49 years, who were either permanent residents or were visitors present in the household on the night before the survey, were eligible to be interviewed. In addition, all children aged 0–59 months who were either permanent residents of the sampled household or visitors present in the household on the night before the survey were eligible to be tested for malaria.

The sample size was determined using 95% confidence limits and a design effect of 1.5 (established based on a similar survey), and 5% adjustment for non-response (from household refusals or abandoned households). To keep the design effect ref as low as possible while maximizing the feasibility of the survey, balance had to be struck between the number of households per cluster (trying to minimize this to reduce the design effect) and the number of EAs (trying to minimize this to reduce the cost, transportation, and workload of the survey teams. Taking both the cost required and the precision to be gained into account, surveying 25 sample households per EA was decided to be optimum.

### Questionnaires and data collection

Two questionnaires were used for the MIS 2016 that is the household and a women’s questionnaire. These were based on the model questionnaires developed by MEASURE DHS + program and adopted and recommended for use by the Roll Back Malaria Monitoring & Evaluation Reference Group (RBM-MERG) Task Force on Household Surveys [5]. They were further adapted for use in Zimbabwe by the MIS Technical Working Group. Both questionnaires were translated into the two main local languages, Shona and Ndebele. The household questionnaire was designed to list all usual members and visitors of the selected households. Some of the basic characteristics of each person collected included: age, sex, religion, education, and relationship to the head of the household. The household questionnaire also identified eligible women to take part in the survey and collected data on household characteristics and assets.

The household questionnaire included questions on IRS, ownership and use of LLINs at household level. The women’s questionnaire was used to collect information from all eligible women aged 15–49 years. The following topics were included:

- Background characteristics, including age and education status.
- Reproductive history and current pregnancy status.
- General malaria knowledge.
- IPTp during recent pregnancies.
- Fever prevalence among children under five and treatment with anti-malarial drugs.

The data collectors used Android-based tablets with CSPro 6.1 based questionnaire, which is an open source application. Data was backed up on SD cards by each data collector periodically as they worked. The same data was then collected and backed up via external hard drives by the teams in the field. The server used was based on File Transfer Protocol (FTP), a standard network protocol used to transfer files between a client and server on a computer network. This local server for the MIS 2016 was setup at the Ministry of Health and Child Care office in Harare. All tablets were linked to the server and data was sent on a daily basis from the field. Data was synchronized at the end of each questionnaire to an FTP server for enhanced security and data protection.

### Assets-based wealth quintiles

The wealth index is a measure that has been used in many Demographic and Health Surveys (DHS) and other country-level surveys to indicate inequalities in household characteristics, in the use of health and other services, and in health outcomes. The index was compiled from the detailed data collected on dwelling and household characteristics and assets using principal components analysis. Table 1 shows the percent distribution of the de jure population (the population that is lawfully part of the household) by wealth quintile according to residence in either moderate or high malaria transmission areas. The table shows that almost twice as many people in the moderate malaria transmission area live in the lowest quintile as those in the high transmission area (29% vs 16%).

**Table 1:** Wealth status

Residence	Wealth quintile					Total	Household members
	Lowest	Second	Middle	Fourth	Highest		
Total	20.0	20.0	20.0	20.0	20.0	100.0	33,716
Moderate	28.6	20.2	16.8	17.3	17.2	100.0	11,623
High	15.5	19.9	21.7	21.4	21.6	100.0	22,093

Percent distribution of household population by wealth quintile, according to residence in malaria transmission level, Zimbabwe MIS 2016

## Results

Table 2 shows that information was collected for just over 33,700 people in the selected households. About 47.8% of the de facto population is male and 52.2% female. The size of the de facto population in the high transmission areas was 1.9 times that found in the moderate transmission areas, reflecting the greater number of high transmission districts in the survey. In general, the proportion of the household population in each age group declines as age increases, reflecting the relatively young average age structure of the population; 44% of the total population was under age 15.

**Table 2:** Household population by age, sex and residence

Age	Malaria transmission								Total
	Moderate				High				
	Male		Female		Male		Female		
	Number	Percent	Number	Percent	Number	Percent	Number	Percent	
< 5	889	16.0	961	15.8	1679	15.9	1757	15.3	5285
5–9	886	16.0	886	14.6	1693	16.0	1702	14.8	5167
10–14	754	13.6	829	13.6	1383	13.1	1435	12.5	4401
15–19	687	12.4	522	8.6	1204	11.4	1068	9.3	3481
20–24	299	5.4	503	8.3	680	6.4	761	6.6	2243
25–29	336	6.1	368	6.1	648	6.1	828	7.2	2180
30–34	345	6.2	463	7.6	721	6.8	885	7.7	2415
35–39	262	4.7	261	4.3	682	6.4	585	5.1	1790
40–44	215	3.9	227	3.7	458	4.3	499	4.3	1399
45–49	163	2.9	144	2.4	294	2.8	254	2.2	855
50–54	105	1.9	199	3.3	228	2.1	382	3.3	913
55–59	122	2.2	159	2.6	202	1.9	368	3.2	852
60–64	105	1.9	154	2.5	210	2.0	296	2.6	765
65–69	77	1.4	136	2.2	156	1.5	277	2.4	646
70–74	61	1.1	66	1.1	107	1.0	125	1.1	359
75–79	45	0.8	51	0.8	90	0.8	105	0.9	290
80+	60	1.1	69	1.1	107	1.0	152	1.3	388
Missing/don’t know	126	2.3	86	1.4	48	0.5	26	0.2	286
Total	5538	100.0	6085	100.0	10,589	100.0	11,504	100.0	33,716

Percentage distribution of the de facto household population by 5-year age groups, according to sex and residence in high or moderate malaria transmission area, Zimbabwe MIS 2016

### Net ownership

Table 3 shows that the percentage of the household population with access to an LLIN was only 13%. Meanwhile, access to an LLIN diminishes steeply as the number of members of the household increases. Those in the higher wealth quintiles also had greater access to an LLIN, but not by that much. Table 3 shows the percentage of the population with access to an LLIN as per the number of sleeping spaces in the household. At least 35–42% of households owned no LLINs at all, regardless of the number of sleeping spaces (Table 4).

**Table 3:** Access to a long-lasting insecticidal net as per number of sleeping spaces

Number of LLINs owned by household	Number of sleeping spaces per household								Number of household members
	1	2	3	4	5	6	7	8+	
0	42.1	38.5	36.2	37.5	42.9	42.0	42.3	34.5	12,983
1	47.6	22.9	18.1	13.6	10.1	11.6	0.0	9.0	7459
2	7.9	31.7	19.2	18.9	13.3	18.2	19.4	20.4	7373
3	1.4	5.0	21.8	14.1	11.2	12.3	16.9	15.6	3821
4	0.3	1.1	3.4	13.8	14.0	4.3	8.8	11.5	1399
5	0.8	0.3	0.8	1.3	5.3	6.5	0.0	3.8	373
6	0.0	0.5	0.4	0.6	1.8	1.0	9.9	3.4	206
7	0.0	0.0	0.0	0.2	0.8	0.0	0.0	0.0	28
8+	0.0	0.0	0.1	0.0	0.4	4.1	2.8	1.8	74
Total	100.0	100.0	100.0	100.0	100.0	100.0	100.0	100.0	33,716

Percentage distribution of households by number of LLINs the household owns, according to number of sleeping spaces in the household, Zimbabwe MIS 2016

**Table 4:** Coverage of malaria testing and prevalence of malaria among children under five

Background characteristic	Children aged 6–59 months				Percent positive by species				Percentage of children aged 6–59 months with positive RDT or blood slide	Number of children aged 6–59 months tested
	Percentage tested for malaria	Number eligible for testing	Percentage positive for malaria RDT	Percentage positive for malaria blood slide	*P. falciparum*	*P. vivax*	*P. ovale*	*P. malariae*		
Total	99.1	2963	0.5	0.2	0.2	0.0	0.0	0.0	0.5	2935
Sex										
Male	99.1	1483	0.6	0.3	0.3	0.0	0.0	0.0	0.6	1469
Female	99.1	1479	0.4	0.2	0.2	0.0	0.0	0.0	0.5	1466
Transmission level										
Moderate	98.7	1024	0.0	0.0	0.0	0.0	0.0	0.0	0.0	1010
High	99.3	1939	0.8	0.3	0.3	0.0	0.0	0.0	0.8	1925
Age										
6–11	99.8	352	0.6	0.4	0.4	0.0	0.0	0.0	0.6	351
12–23	99.5	730	0.4	0.1	0.1	0.0	0.0	0.0	0.4	727
24–35	99.0	635	0.0	0.2	0.2	0.0	0.0	0.0	0.2	629
36–47	99.1	740	0.9	0.4	0.4	0.0	0.0	0.0	0.9	734
48–59	97.9	505	0.6	0.1	0.1	0.0	0.0	0.0	0.6	494
Wealth quintile										
Lowest	98.5	573	0.4	0.4	0.4	0.0	0.0	0.0	0.6	565
Second	99.2	604	1.2	0.4	0.4	0.0	0.0	0.0	1.2	599
Middle	99.0	554	0.5	0.0	0.0	0.0	0.0	0.0	0.5	548
Fourth	98.9	521	0.4	0.1	0.1	0.0	0.0	0.0	0.4	515
Highest	99.6	711	0.2	0.2	0.2	0.0	0.0	0.0	0.2	708

For children 6–59 months eligible for malaria testing, percentage who have been tested, with prevalence of malaria, by background characteristics, Zimbabwe MIS 2016

### Indoor residual spraying

Table 5 shows that 62% of households in moderate and high transmission zones had had IRS in the 12 months preceding the survey. Slightly more households had been sprayed in the high malaria transmission areas than in moderate transmission areas—64% and 60%, respectively. According to the figures above, the government had undertaken the IRS in the overwhelming majority of households sampled (96%); less than 2% of the spraying had been carried out by private organizations.

**Table 5:** Household availability of insecticide-treated nets and protection by IRS among all households sampled

Percentage of households with at least one mosquito net (untreated or LLIN), one mosquito net per sleeping space (untreated or LLIN), and households with at least one LLIN and/or IRS in the last 12 months, by background characteristics and malaria transmission level, Zimbabwe MIS 2016											
Percentage of households with at least one mosquito net					Percentage of households with at least one net per sleeping space						
Background characteristic	No mosquito net	Any mosquito net	Any other net [untreated or insecticide-treated net (ITN)]	LLIN	Any mosquito net	LLIN	Any other net (untreated or ITN)	Percentage of households with IRS in the past 12 months	Percentage of households with at least one LLIN and/or IRS in the last 12 months	Percentage of households with at least one LLIN per sleeping space and/or IRS in last 12 months	Number of households
Total	38.5	61.5	4.4	58.1	53.8	50.7	4.4	62.4	85.3	82.5	8026
Sex of household head											
Male	38.8	61.2	4.3	58.0	52.5	49.7	4.3	63.6	86.0	82.3	5212
Female	37.9	62.1	4.7	58.2	56.2	52.5	4.7	60.2	84.1	82.9	2815
Malaria transmission level											
Moderate	36.2	63.8	2.9	62.0	57.1	55.2	2.9	59.8	82.8	80.7	2543
High	39.6	60.4	5.1	56.2	52.3	48.6	5.1	63.6	86.5	83.4	5484
Wealth quintile											
Lowest	43.4	56.6	3.5	53.7	48.1	45.4	3.5	74.9	88.2	86.2	1380
Second	46.7	53.3	3.4	50.6	46.4	43.8	3.4	73.2	86.6	84.9	1473
Middle	40.6	59.4	5.4	55.3	52.3	48.8	5.4	76.8	89.3	88.0	1463
Fourth	37.1	62.9	4.3	59.9	54.2	51.8	4.3	69.9	86.4	84.5	1542
Highest	29.4	70.6	5.0	66.5	63.3	59.3	5.0	32.2	79.3	73.5	2169

### Knowledge of causes of malaria

The vast majority of respondents (85%) knew that mosquitoes spread malaria, and that sleeping under a mosquito net can protect against getting malaria (80%). But there were still some serious misconceptions about the disease, with 14% stating that they thought malaria was caused by dirty water, and 5% blaming watermelons and sugarcane.

When asked about the danger signs of malaria in children, once again, only 50% of respondents identified fever as one of the major signs. And only a very low 13% mentioned seizure and convulsions. There was some minor variation in knowledge with regard to wealth quintiles and age of respondent, but not much. Table 6 shows that only 35% of respondents identified high fever as a malaria danger sign in adults, and 18% seizures and convulsions.

**Table 6:** Knowledge of causes of malaria

Percentage who reported specific causes of malaria							
Background characteristic	Mosquito bites	Dirty water	Watermelon/sugarcane	Harmful spirits	Other	Don't know	Number of respondents
Total	84.7	14.2	5.4	0.1	7.1	4.4	8026
Head of household							
Male	86.1	14.3	5.7	0.2	6.9	3.4	5212
Female	82.0	14.0	4.9	0.1	7.5	6.3	2815
Transmission level							
Moderate	80.9	16.2	5.4	0.1	8.8	6.7	2543
High	86.4	13.3	5.4	0.2	6.4	3.4	5484
Age							
15–19	83.5	13.0	1.5	0.7	4.2	7.1	85
20–24	84.5	8.3	4.3	0.0	5.5	8.3	356
25–29	88.4	9.8	6.9	0.0	10.2	1.5	877
30–34	90.3	9.8	3.7	0.0	2.9	5.8	1272
35–39	89.4	14.5	5.3	0.2	7.5	2.1	1017
40–44	88.6	13.1	5.1	0.0	7.1	2.0	852
45–49	87.0	15.9	4.7	0.3	7.5	1.5	590
50–54	85.9	14.0	5.3	0.1	5.6	3.0	590
55–59	82.0	18.0	5.6	0.2	9.5	3.5	552
60–64	82.0	19.3	7.3	0.2	8.9	10.2	525
65–69	71.7	20.7	4.8	0.0	7.2	4.7	475
70–74	69.0	21.7	7.6	1.0	11.6	8.6	270
75–79	72.8	18.4	7.8	0.3	8.9	7.8	214
80–84	65.2	19.5	10.3	0.0	10.7	9.3	134
85+	67.7	18.7	8.4	0.0	5.4	9.5	119
Missing	82.6	7.4	2.3	0.0	3.8	10.5	97
Wealth quintile							
Lowest	79.2	15.7	7.4	0.2	8.4	5.5	1380
Second	83.5	15.3	6.6	0.3	7.2	4.2	1473
Middle	83.9	17.2	5.6	0.1	6.3	3.6	1463
Fourth	83.4	16.1	4.9	0.0	7.9	2.6	1542
Highest	90.3	9.2	3.6	0.1	6.3	5.8	2169

Percentage distribution of household members who identified specific causes of malaria in the community, by background characteristics, Zimbabwe MIS 2016

## Discussion

IRS is the spraying of the interior walls of a dwelling with an insecticide and has been a mainstay of malaria vector control in Zimbabwe for many years. It reduces the transmission of malaria by killing adult female mosquitoes when they rest on the walls of the dwelling after feeding [6]. Households are considered to be covered by a vector control intervention if they own at least one LLIN and/or they have been sprayed by IRS at any time in the past 12 months. Malaria Indicator Survey 2016 found that 62% of households possessed a mosquito net, with 58% owning an LLIN. Slightly more households in the moderate malaria transmission areas owned an LLIN (62%) compared to those in the high malaria transmission areas (56%). Just under two-thirds of households had received IRS in the 12 months preceding the survey, with slightly more households sprayed in the high malaria transmission areas compared to in the moderate transmission areas—64% and 60%, respectively. Eighty-three percent of households had at least one LLIN per sleeping space, and/or had received IRS over the last 12 months.

There appears to be a general improvement in the overall ownership of LLINs from 2012 (46%) to 2016 (58%), although these figures are still below the target of universal access to nets [7, 8]. Similarly, there was a 55% increase in IRS coverage over the reporting period (2012, 49%; 2016, 62%) as shown in Table 7. The overall protection coverage by at least one major vector control measure, IRS and/or LLINs, was high 58%. Fifty-four percent of the sampled population had slept under a net the night before the survey. Only a third of children aged under 5 years had slept under an LLIN the night before the survey. However, protection by at least one vector control intervention was significantly higher, as 74% had either slept under an LLIN the previous night or slept in a dwelling that had received IRS in the past 12 months. About a third of women aged 15–49 years had slept under an LLIN the previous night, compared to only a quarter of pregnant women.

**Table 7:** Progress on key malaria indicators to date

Indicator	2010–2011 ZDHS^1^	2014–2015 ZDHS^2^	2012 MIS^3^	2016 MIS^4^
Proportion of the population that slept under a net the night before the survey	NA	NA	NA	54%
Proportion of households that own at least one LLIN	25%	47.9%	46%	58%
Proportion of children under five who slept under an LLIN the night before the survey	8%	9.0%	50%	32.5%
Proportion of women 15–49 who slept under an LLIN the night before the survey	8%	6.1%	49%	36%
Proportion of pregnant women sleeping under an LLIN the night before the survey	9%	13.1%	NA	24.5%
Proportion of pregnant women who received at least two doses of IPTp, with at least one dose during antenatal care (ANC)	7%	N/A	35%	37%
Proportion of households with IRS in the past 12 months	17%	N/A	49%	62%
Prevalence of parasitaemia (by microscopy) in children 0–59 months	NA	NA	0.4%	0.2%
Prevalence of parasitaemia (by microscopy) all age groups	NA	NA	NA	0.2%

^1^406 EAs in all 10 provinces surveyed, including non-malarious districts

^2^400 EAs in all 10 provinces surveyed, including non-malaria districts

^3^MIS conducted in 327 EAs in 51 districts, collecting data on LLINs only in 30 targeted districts; on IRS only in 45 targeted districts; and on IPTp only in 30 targeted districts

^4^MIS conducted in 353 EAs in 45 moderate and high-risk malaria districts, without disaggregation by type of intervention (LLINs, IRS, IPTp)

Forty-two percent of women either had taken two doses of sulfadoxine-pyrimethamine (SP)/Fansidar or were on lifelong co-trimoxazole during pregnancy. This IPTp coverage has remained almost unchanged since the 2012 MIS, with only a third of pregnant women receiving at least two doses of IPTp. Microscopic analysis confirmed that *Plasmodium falciparum* remains the predominant parasite species in the country. Twenty percent of children under 5 years of age had experienced an episode of fever in the two weeks prior to the survey, with two-thirds of caretakers having sought treatment for their child when this happened. Also, 12% of women aged 15–49 years reported that they needed permission from someone else before seeking medical care for a child under five with fever. This parallel finding of the Zimbabwe DHS 2010–2011, in which 16% of the women respondents reported that they do not make their own decisions about their own health care.

Malaria prevalence was assessed in all age groups. Among children under 5 years of age, 0.5% were found positive for malaria by rapid diagnostic test (RDT) and 0.2% by microscopy; for children aged 5–14 years the prevalence rate was 0.8% by RDT and 0.2% by microscopy; while among those aged 15 years and above, 0.5% were positive for malaria by RDT and 0.3% tested malaria slide-positive. There was a notable decline in exposure to malaria messages despite relatively high ownership, among the households sampled, of radios, TVs, and mobile phones. This also correlates with a decline in knowledge of the symptoms and danger signs of malaria compared to respondents’ reports in the 2012 MIS. The 2016 Malaria Indicator Survey seems to suggest that there are still challenges in seeking treatment as less than half of the households sampled were within a 5-km radius of a health facility. Furthermore, the other concern is that some women still report needing permission from some other person to bring a child with fever to a health facility for treatment. Only a quarter of the women interviewed reported that they would stop treatment for malaria when the child had taken the full dose prescribed by the health worker. There is need for continued mobilization and advocacy to the communities to ensure that malaria is put as priority at family level, eliminating a need for permission which may continue to co complicate malaria symptoms [9, 10].

## Conclusion

Prevalence of malaria declined from 0.4% in 2012 to 0.2% in 2016 for children below 5 years which shows good direction towards malaria elimination targets. The prevalence for the age group 5 years and above was 0.2% although no comparative figure was available for 2012. There has been a general improvement in the overall ownership of LLINs from 2012 to 2016, although these figures are still below the target of universal access to nets. This therefore pose a challenge in view of the investment in these interventions and the malaria elimination goals [10]. On the other hand, there was a notable increase in IRS coverage over the reporting period. This could account for the fact that protection by at least one vector control method, IRS and/or LLIN, surpassed the programme target in 2016. Of concern though is the reported low net utilisation among high-risk groups such as children under 5 and pregnant women, despite the general increase in net ownership. Moreover, IPTp coverage has remained almost unchanged since the 2012 MIS with only a third of pregnant women receiving at least 2 doses of IPTp.

There is a need to expand continuous net distribution so that nets are replaced between the mass LLIN distribution campaigns. This will cover the attrition gap and ensure sustained universal coverage of nets across the communities. There should be strengthened SBCC campaigns, especially for delivering messages that foster mosquito net use in high-risk populations such as young children and pregnant women. It is important to undertake regular, local assessments of net use and take necessary remedial actions to increase and maintain net use. Information, education, and communication on malaria and its prevention needs to be strengthened [11]. With the high mobile phone penetration of the country, the NMCP should consider using this cost-effective platform more often to increase dissemination of malaria messages. These messages should focus on the danger signs of malaria; the importance of seeking treatment early, as well as compliance with the full course of malaria treatment; and the importance of using an LLIN at all times, even in what is not supposed to be the malaria transmission season [12].

## Data Availability

All data is available if further information is required.

## Abbreviations

ANC: antenatal care
CHAI: Clinton Health Access Initiative
CSPro: census and survey processing system
EA: enumeration areas
IPTp: intermittent preventive treatment in pregnancy
IRS: indoor residual spraying
LLINs: long-lasting insecticidal nets
MICS: Multiple Indicator Cluster Surveys
NIHR: National Health Institute of Research
NMCP: National Malaria Control Programme
PMD: Provincial Medical Directors
PSI: Population Services International
RBM-MERG: Roll Back Malaria Monitoring and Evaluation Reference Group
RDT: rapid diagnostic test
SBCC: Social and Behaviour Change Communication
SP: sulfadoxine/pyrimethamine
WHO: World Health Organization
ZAPIM: Zimbabwe Assistance Program in Malaria
ZDHS: Zimbabwe Demographic and Health Surveys
ZIMSTAT: Zimbabwe National Statistics Agency
ZMIS: Zimbabwe Malaria Indicator Survey
ZMS12: Zimbabwe Master Sample 2012
