# Correction to: How far is the journey before malaria is knocked out in Zimbabwe: results of the malaria indicator survey 2016

**DOI:** 10.1186/s12936-019-2823-x

**Published:** 2019-06-12

**Authors:** Busisani Dube, Joseph Mberikunashe, Patience Dhliwayo, Andrew Tangwena, Gerald Shambira, Anderson Chimusoro, Munashe Madinga, Brighton Gambinga

**Affiliations:** 1 National Malaria Control Programme, Harare, Zimbabwe; 2 University of Zimbabwe, College of Health Sciences, Harare, Zimbabwe; 3 World Health Organization, Country Office, Harare, Zimbabwe; 4 Clinton Health Access Initiative, Country Office, Harare, Zimbabwe

## Correction to: Malar J (2019) 18:171 10.1186/s12936-019-2801-3

Following publication of the original article [1], the authors of the article flagged that their article had gone to publishing with an error in the title.

The incorrect title read: “How far is the journey before malaria is knocked out malaria in Zimbabwe: results of the malaria indicator survey 2016”.

The correct title, however, reads: “How far is the journey before malaria is knocked out in Zimbabwe: results of the malaria indicator survey 2016”.

The title has been corrected in the published article.

The publisher apologizes for this processing error.
